# Sustainable choices: The relationship between adherence to the dietary guidelines and food waste behaviors in Italian families

**DOI:** 10.3389/fnut.2022.1026829

**Published:** 2022-12-14

**Authors:** Federica Grant, Laura Rossi

**Affiliations:** Council for Agricultural Research and Economics-Research Centre for Food and Nutrition (CREA Food and Nutrition), Rome, Italy

**Keywords:** food waste behaviors, dietary recommendations, eating habits, sustainability, household, Italy

## Abstract

**Introduction:**

Food loss and waste are urgent problems to address. Recent estimates highlighted that the highest quantities of waste are generated at the household level and for this reason, the interest in this sector has increased over years.

**Methods:**

To investigate if there is a connection between consumers’ behaviors aiming at reducing food waste and consumers’ choices in adopting healthy eating habits, a survey among a sample (*n* = 2,869) representative of the Italian population was carried out with the use of validated questionnaires.

**Results:**

Results demonstrated that the higher the adherence to the Italian dietary guidelines indicator (AIDGI) the higher the score measuring household food waste behaviors (HFWB). In particular, the highest AIDGI corresponds to a preponderance of respondents that was more able to plan the shopping and the use of food (38.9%, *p* < 0.001), to better evaluate the quantities to cook (40.4%, *p* < 0.001), to avoid impulsive buying (35.2%, *p* < 0.01), to have a high knowledge of the food stored (38.4%, *p* < 0.001), to reuse leftovers (35.4%, *p* < 0.001), to assess food safety (34.7%, *p* < 0.001), to plan accurately (34.9%, *p* < 0.01), to know how to prolong the shelf life of a product (34%, *p* < 0.05), and to cook creatively (32%, *p* < 0.01). In addition to that, half of the respondents with the lowest AIDGI score did not receive any education regarding food waste (51.1%, *p* < 0.001). HFWB indicators globally resulted in scores ranging from 40 to 80% revealing the attention of Italians to food waste issues. Regarding eating habits, in half of the sample (50.4%) a consumption pattern with low adherence to nutritional recommendations was found, in particular among men (34.4%), younger age groups (40%), and people living in large families (42.3%).

**Discussion:**

The overall results provided interesting information that could give input for planning nutrition education actions and identifying targets and topics to be addressed.

## 1 Introduction

Food loss and waste (FLW) is a problem that needs to be addressed urgently due to its social, economic, and environmental implications. The main sector responsible for generating waste along the food supply chain is the household consumption level. According to recent estimates, 17% of available food is wasted globally, with 61% of which consisting of household food waste (1). Various studies have highlighted that household food waste is influenced by specific causes and determinants (2–6). In 2018, a research model was developed to categorize the reasons for household food waste (7, 8), identifying Motivations, Opportunities, and Abilities (MOA) as the main drivers of food waste. The motivations include awareness of food waste and the social norms related to throwing away food. The opportunities consist of the access to grocery shops either as typology and variety of products, or shop organization, e.g., the opening times and the geographical proximity. The abilities concern all the factors related to the organizational aspects of eating, such as planning, storing, and cooking the food. Although not included in the classical MOA models, the researchers also looked at other potential drivers of FLW, including whether education received from parents on food waste had an impact on the amount of food thrown away (9, 10). All these factors can influence household food waste prevention practices, which can be identified as planning the shopping and the use of the food, avoiding impulsive buying, checking for food already stored, cooking the right quantities of food for the family, and storing or using leftovers (7).

The MOA theoretical framework on household food waste was used in Italy in a national survey carried out in 2018 (11), to investigate the food waste behavioral profile of Italian consumers to obtain data to address the causes and to design FLW prevention strategies. In Italy, throwing away food is associated with a widespread negative emotional experience, with the majority of respondents stating that they considered food waste a deplorable practice. For Italian consumers, the ethical aspects of food waste are more important than the ecological consequences. At the household level, time availability and unexpected events were reported as key aspects of difficult food management in the kitchen, even though respondents declared abilities in the use of the leftovers.

The 12th goal of the sustainable development goals (SDGs) of the 2030 agenda established by the United Nations (12, 13) regarding sustainable production and consumption patterns includes target 12.3 which focuses on halving per capita food waste at the retail and household level by 2030 (14). Adopting a sustainable diet could be a strategy to limit the environmental impact of the food system and to make consumers more sensitive to the FLW problem (15, 16). Several studies have stated that the Mediterranean diet, which has been largely recommended for many years for its health-protective aspects, is also sustainable (17). The Mediterranean diet pattern has a low impact on soil, water, and energy resources (18). The Mediterranean diet principles were followed to establish the Italian food-based dietary guidelines, which were updated in 2018 with a focus on the sustainability of the dietary pattern. In particular, directive no. 13 of the Italian dietary guidelines brings together recommendations on how to adopt a sustainable lifestyle that can improve the quality of the diet and that can reduce food waste (19).

The rationale of this work is based on the recommendations of the Europe sustainable development report 2021 (20) that underlies the need for European union member states, including Italy, to adopt significant actions to achieve the 12th goal of SDGs to fulfill what the 2030 agenda established. In this sense, the analysis and knowledge of food waste behaviors and their relationship with healthy eating habits may be considered determinant elements to pursue the sustainability goal. However, data on these topics are limited and with non-univocal findings. Helander et al. (21) reported that a shift toward a healthy and sustainable diet can lead to an increased amount of food waste considering that a healthy diet is characterized by the consumption of products that largely contribute to food waste, such as fruit, vegetables, and milk. To the best of our knowledge, the relationship between the determinants of a healthy diet and food waste is a new area of interest investigated only in a few studies. Conrad et al. (22) found that high-quality diets were associated with greater food waste and Carroll et al. (23) reported a correlation between diet quality and fruit and vegetable waste. Similar results were reported in the study of Mijares et al. (24), which observed that the quantity of waste of fresh vegetables, cereals, and dairy products was related to a higher quality of the diet even though the high quality of diet was associated with low total food waste. On the same topic, another study pointed out that consumers who pay particular attention to food consumption and nutrition have also attitudes to prevent and limit food waste generation confirming the idea that healthy eating habits are associated with a sustainable lifestyle (25). In consideration of this scenario, we would demonstrate that attention to sustainability issues can affect both eating habits and food waste behaviors.

The main purpose of this study is to evaluate the consumers’ food waste behaviors and their food habits investigating whether there is a connection between these two aspects. The hypothesis underlying this research is that adherence to dietary recommendations is linked to a good food management capacity and consequently to the prevention of food waste at the household level. This work would address the following research questions: (i) to what extent the adherence to dietary recommendations is related to food waste attitudes? (ii) To what extent do sociodemographic aspects influence consumers’ behaviors in terms of dietary patterns? (iii) Nutrition educational activities could be the place to promote food waste prevention practices?

This work is part of the activities of the Italian Observatory on food surplus, recovery, and waste, a technical entity with a pivotal role in the production of research, methodologies, and reliable data that can be used as drivers for policy actions. One of the priorities recognized by the Observatory was to assess and monitor household food waste at the national level to support the development of actions aimed at reducing the amount of food waste (26). In light of this commitment, the behavioral assessment presented in this paper will provide inputs to allow for a better understanding of the causes of household food waste, as well as information to develop potential targets and intervention strategies to help reduce waste in the framework of the promotion of healthy diet.

## 2 Materials and methods

### 2.1 The survey methodology

A cross-sectional survey including 2,869 respondents, representative of the Italian adult population (age > 18 years), was carried out.

The data collection was performed by SWG S.p.A., a specialized market research agency, through interviews carried out among a panel group, including more than 60,000 individuals profiled according to the main sociodemographic variables and purchasing habits. The online procedure through the computer assisted web interviewing (CAWI) method was self-completed by 2,619 participants. The remaining sample (*n* = 250) consisted of people who were unfamiliar with the online system and therefore used the computer assisted personal interviewing (CAPI) method, with direct contact with the operators. The sampling plan was carried out to provide a stratification for area of residence and using simultaneously fixed quotas for age classes and gender. During the survey, the number of key component parameters such as the family size and the level of education were kept under control. The sample size of 2,500 was calculated in order to cover 11 territorial areas with a probability proportional to size methodology and with a statistical margin of error of 1.82% at 95% of confidence interval. The reported sampling permitted to cover Italian macro-regions and some high-density population areas in order to assess the territorial variability related to socioeconomic and cultural diversity between the Italian regions. The sample size was increased of 10% to cover the population that do not use internet. All indicators were aligned with the data provided by istituto nazionale di statistica (ISTAT) related to 2020 (27). The data were weighted to ensure the representativeness with respect to the parameters of area, gender, age, and level of education.

To participate in the SWG panel consumer surveys, respondents were required to sign a privacy agreement and consent form to collect and process their personal data in advance, following the Italian data protection law (Legislative Decree 101/2018) in line with European commission general data protection regulation (679/2016). Before starting the data collection, participants were informed about the objective of the research and the consequent statistical analysis. Participation in the study was fully voluntary and anonymous and subjects could withdraw from the survey at any time and for any reason. This study was conducted according to the guidelines of the declaration of Helsinki (28) and all procedures involving research study participants were approved and are in line with the SWG code of conduct (29). The assessment did not involve any invasive procedures nor induce any changes in dietary patterns. Therefore, the study did not require approval from the ethics committee.

The data collection was performed between the 26th of June and the 20th of July 2020. This period was selected in consideration of the fact that the social restrictions related to the Coronavirus disease-2019 (COVID-19) pandemic in Italy were attenuated from the 18th of May 2020 and further reduced after the 3rd of June 2020 when all the social activities started again and free movement between regions was allowed.

### 2.2 The survey structure and the questionnaire

The measurements carried out in the present study were shaped according to the objective of the survey. An articulated questionnaire was administrated with the first part covering sociodemographic information (gender, age, region of residence, education, job, income, and family size). Two main modules constitute the core of the assessment tool: (i) the household food waste behaviors (HFWB) questionnaire; (ii) a food frequency questionnaire that permitted the evaluation of adherence to the Italian dietary guidelines indicator (AIDGI). These two modules represented the capitalization of the work carried out in previous studies (11, 30) in which the methodologies of data collection were tested, validated, and adapted to the Italian context. The full questionnaire used for this paper is reported in the Supplementary material (Supplementary Table 1).

Household food waste behaviors were measured with the validated questionnaire developed by van Herpen et al. (31) and further adapted to the Italian context (11) assessing determinants and behaviors of consumers toward food waste. The HFWB module included 39 questions that assessed (i) prevention practices (planning the shopping and using the food, avoiding impulsive buying, the overview of stored food, cooking the right quantities of food, and storing and using leftovers), (ii) abilities (the perceived difficulty with assessing food safety, the perceived difficulty with cooking creatively, the perceived difficulty with accurate planning, and the knowledge of prolonging the shelf-life), and (iii) education received from parents (parents’ attention to prevent food waste). A 7-point scale was used with answers ranging from “strongly disagree” to “strongly agree” or from “never” to “always.” The answer scales were further grouped into four categories following the quartile distributions that varied for each behavior related to food waste: low, low–medium, medium–high, high.

Eating habits were evaluated by adapting the food frequency questionnaire used by the Italian national institute of statistics (27). The module consists of 18 food category items. For each category respondents were asked to quantify the frequency of consumption on a scale of five possible answers: more than once a day, once a day, few times a week, less than once a week, never. Hence, the AIDGI was created with a procedure similar to Benedetti et al. (32). AIDGI was based on a qualitative frequency scale and provided a synthetic evaluation of the adherence to a healthy diet defined in the dietary guidelines. For each food group, the following scores were assigned: +2 points in case of frequency of consumption in line with recommendations, 0 points in case of frequency of consumption very far from recommendations, and +1 points for answers close to the recommendations, but not exactly in line with them. AIDGI was calculated as the sum of 18 group scores. For example, for the groups “fresh fruit” and “vegetables,” the maximum score (2 points) was set for “more than once a day,” a score of 1 was assigned to the option “once a day,” and 0 scores were assigned to the other reported intakes. The scores obtained from each category were summed up and four AIDGI levels were identified: low (0–18), medium–low (19–20), medium–high (21–23), and high (> 23).

### 2.3 Data analysis

Descriptive statistics to illustrate the most important characteristics of the data collected were performed, such as food waste behaviors, the food habits of Italian consumers, and the adherence to nutritional recommendations. AIDGI and HFWB ordinal measures were calculated based on quartiles of the quantitative scores. A contingency analysis was performed to check associations between variables such as AIDGI and sociodemographic, and AIDGI and HFWB. Specifically, double-entry tables were processed, and the Chi-squared test of independence was applied along with *post-hoc* tests to check pairwise comparisons with Bonferroni corrections of the *p*-values. A *p*-value less than 0.05 was fixed for statistical significance. The statistical analysis was performed using IBM SPSS Statistics, version 25.

## 3 Results

### 3.1 Sociodemographic characteristics of the sample

The study sample resulted aligned to the Italian socio-demographic composition (27) for the effect of the sampling procedure and the subsequent weighting of the data. In particular, variables such as gender, age, and region of living have the same distribution of the Italian population. Small differences were observed amongst the level of education and the family size. In the present sample, the medium and high level of education are more represented (respectively +6 and +4%) than in the Italian population while the low level of education is less common (−9%) respect to the percentages measured at the national level. For the family size, single-member families are lower represented (−18%) and two-members families are more represented (+7%) respect to Italian population. Nevertheless, the overall distribution of smaller (one and two members) and larger (more than three members) families is similar to the national data. The results are therefore representative of the population in Italy, distributed in macro-regions corresponding to the local population density (Supplementary Table 2).

### 3.2 Household food waste behaviors in Italy

#### 3.2.1 Prevention practices

**FIGURE 1:**
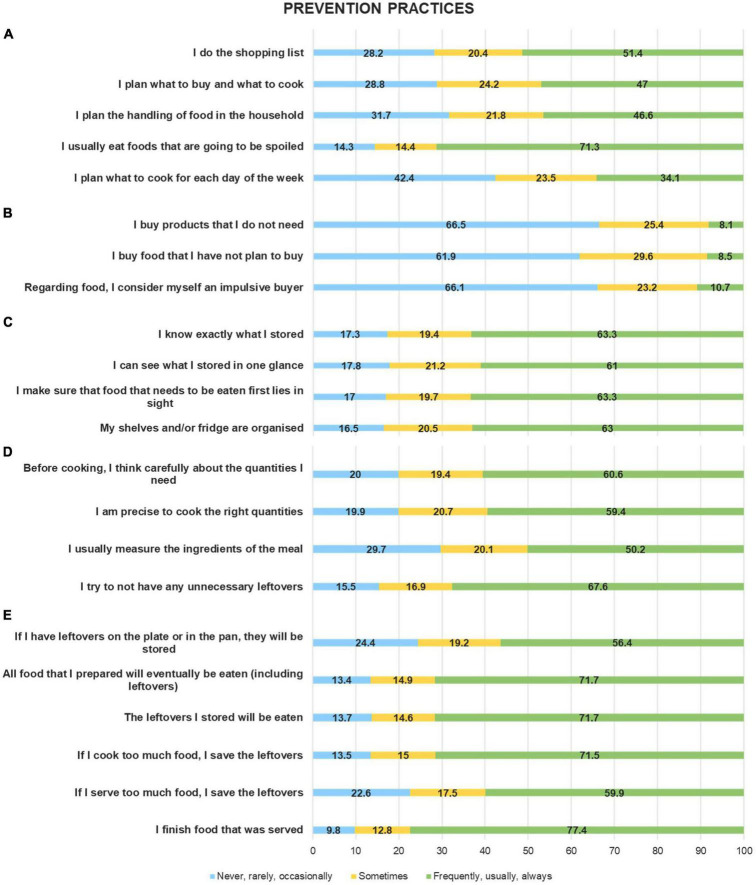
Food waste prevention practices among the sample: planning the shopping and using the food **(A)**; avoiding impulsive buying **(B)**; the overview of stored food **(C)**; cooking the right quantities of food **(D)**; storing and using leftovers **(E)**. Percentage values (%).

Figure 1 reports the results obtained from the survey on household food waste prevention practices that included five subsets of questions (panels A, E). Planning food shopping and handling food in the household were common practices. More than two–thirds (71.3%) of the Italian families in the sample paid attention to eat purchased foods before throwing them away. Around half of the respondents, declared they made a food shopping list, planned what to buy and what to cook, and organized the management of food in the family. Planning what to cook for the week was less frequently reported (34.1%) (Figure 1, panel A). The majority of Italian families (66.1%) did not consider themselves impulsive buyers, avoiding buying no-needed or no-planned products. However, around 25% of the sample declared having bought “sometimes” unnecessary products (Figure 1, panel B). Having a good overview of the food inventory in the kitchen, as well as knowing what was in the fridge or stored in the pantry, were practices commonly adopted by Italian families, with approximately 60% of respondents having reported the habit of checking the quality and the quantity of stored food (Figure 1, panel C). Regarding the precision in cooking (Figure 1, panel D), avoiding producing leftovers was reported by around 70% of respondents, alongside the ability to be precise in measuring ingredients, preparing, and cooking the correct amount of food for the families. Italian families were also careful to eat all food that was prepared, including leftovers (Figure 1, panel E). The majority of respondents (77.4%) finished all the food on their plates. If there were leftovers (either due to having cooked or served more than what was necessary) they were stored and further reused.

#### 3.2.2 Abilities

**FIGURE 2:**
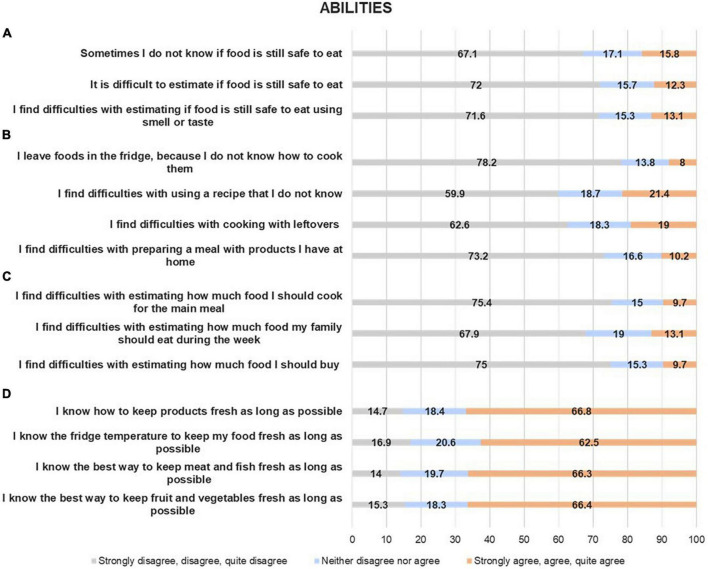
Food waste abilities among the sample: the perceived difficulty with assessing food safety **(A)**; the perceived difficulty with cooking creatively **(B)**; the perceived difficulty with accurate planning **(C)**; the knowledge of prolonging the shelf-life **(D)**. Percentage values (%).

In Figure 2, the results of the subset of questions related to the influence of the consumers’ abilities on food waste generation are reported.

Around 70% of Italian families had no difficulties with understanding whether the food was still edible and safe to eat, using smell or taste, or evaluating the external aspect (Figure 2, panel A).

More than 60% of the respondents reported having cooked creatively, using the food that was in the fridge and leftovers, and experiencing new recipes to cook (Figure 2, panel B).

Concerning food purchase planning, around 70% of respondents declared they were able to estimate the quantities of foods to buy and cook, to satisfy the needs of the family (Figure 2, panel C).

Panel D of Figure 2 reports the answers to the questions related to the knowledge of prolonging the shelf-life of foods. Around 60% of respondents considered themselves to know the best way and the optimal temperature to store fresh foods (fruit, vegetables, meat, and fish) and preserve them longer.

#### 3.2.3 Education received from parents

**FIGURE 3:**
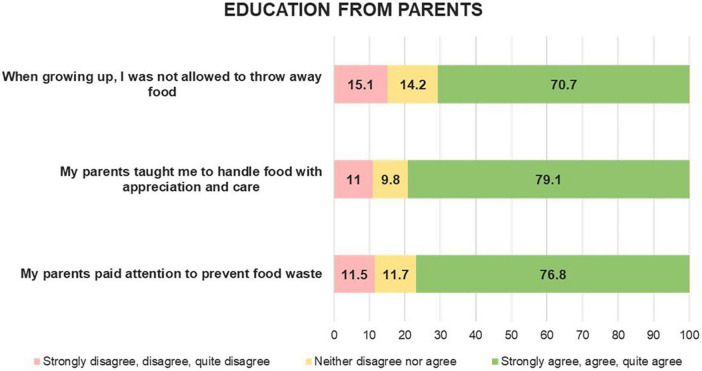
Education received from parents among the sample. Percentage values (%).

Education received from parents was reported as a factor impacting on food waste behavior for almost 80% of Italian families (Figure 3). The attention of the parents to food waste and being taught not to throw away food were reported as having influenced the respondents’ attitudes to food waste early in their childhood.

### 3.3 Adherence to the Italian dietary guidelines

Italian consumers did not follow the dietary guidelines as far as concerning the intakes of processed meat, (69.9%), sugary drinks (74.7%), and alcoholic beverages (80.4% beer and wine, 63.9% other alcoholic drinks) which resulted higher than recommended. In addition, most of the respondents (90%) reported a frequency of consumption of milk and yogurt lower than the recommendations. Bread, pasta, and rice were consumed in the adequate quantities only in 20% of cases, although around 50% of families declared a consumption frequency near the recommendations. However, most of the respondents reported to follow the guidelines in terms of the occasional consumption of cakes and sweets (80.5%) and savory snacks (57.2%). Almost half of the families reported an appropriate consumption of legumes (55.9%), nuts (51.9%), dairy products (57.8%), fish and fisheries products (55.4%), and white meat (68.3%). In line with dietary guidelines, a low intake of red meat was declared by 52.4% of respondents while a high percentage of them reported an appropriate level of consumption of fruit (40.7%) and vegetables (29.8%).

These results are reported in Supplementary Table 3.

### 3.4 The AIDGI and Italian sociodemographic variables

The AIDGI was conceived to identify four levels of adherence to nutritional recommendations that in our sample were homogeneously distributed: 28.9% low; 21.5% low–medium; 25.5% medium–high; 24.1% high. The relationship between the AIDGI and sociodemographic variables (Supplementary Table 4) showed that among those who obtained the lowest AIDGI score, a preponderance of men (*p* < 0.001), younger groups (18–44 years old, *p* < 0.01), and families with five or more members (*p* < 0.05) was found. On the other hand, in the highest AIDGI level group, a preponderance of women (*p* < 0.05), older groups (≥ 55 years old, *p* < 0.001), and families with two members (*p* < 0.05) was observed. No significant differences were observed concerning education level and income groups (data not shown).

### 3.5 The relationship between AIDGI and the adoption of household food waste behaviors

The results of the contingency analysis with the Chi-square test between AIDGI and HFWB are shown in Table 1. In the group of participants with the highest adherence to dietary guidelines there was a significant preponderance of respondents that was more able to plan the purchasing and the use of food (38.9%, *p* < 0.001), to better evaluate the quantities to cook (40.4%, *p* < 0.001) and to avoid impulsive buying (35.2%, *p* < 0.01). In addition, in this group a medium–high and high knowledge of the food stored (respectively 31.2 and 38.4%, *p* < 0.001) and a tendency to reuse leftovers (respectively 30.5 and 35.4%, *p* < 0.001) were observed. On the other hand, more than one–third of Italian families who obtained the lowest AIDGI scores reported a less frequent adoption of food waste prevention practices (*p* < 0.001).

**TABLE 1:** The relationship between adherence to the Italian dietary guidelines indicator (AIDGI) and household food waste behaviors (HFWBs) (prevention practices, abilities, education received from parents).

		AIDGI levels			
Overall sample		Low (%) 28.9	Low–medium (%) 21.5	Medium–high (%) 25.5	High (%) 24.1
**Prevention practices**					
Planning and using	Low (0–3.6)	44.8*	20.4	19.2	15.7
	Low–medium (3.7–4.4)	28.3	28.4	25.5	17.7
	Medium–high (4.5–5.4)	21.2	20.6	31.5	26.7
	High (>5.4)	16.6	16.9	27.6	38.9*
No impulsive buying	Low (1–4.3)	36.5*	22.4	24.8	16.4
	Low–medium (4.4–5.0)	29	22	25.2	23.7
	Medium–high (5.1–6)	27.5	21	25.5	25.9
	High (>6.0)	17.5	19.9	27.4	35.2*
Overview of stored food	Low (1–4.0)	50.2*	22	17.2	10.6
	Low–medium (4.1–5.0)	22.8	23.7	32.5	21
	Medium–high (5.1–5.75)	19.3	21.5	28	31.2*
	High (>5.75)	18.4	18.1	25	38.4*
Cooking the right quantities	Low (1–4.0)	45.6*	25	18.2	11.3
	Low–medium (4.1–5.75)	24	23.9	29.1	23
	Medium–high (4.75–5.75)	21.9	19.7	32	26.3
	High (>5.75)	17.1	16.8	25.7	40.4*
Using leftovers	Low (1–4.17)	50.5*	21.5	16.6	11.5
	Low–medium (4.18–5.33)	27.9	26.1	26.3	19.7
	Medium–high (5.34–6.35)	19.2	20.3	30	30.5*
	High (>6.35)	16.9	18.1	29.6	35.4*
**Abilities**					
No difficulty with assessing food safety	Low (1–4.34)	38.5*	25.9	20.1	15.6
	Low–medium (4.35–5.67)	35.4*	18.7	26.4	19.5
	Medium–high (5.68–6.67)	23.3	18.8	29.1	28.8*
	High (>6.67)	15.8	21.8	27.7	34.7*
No difficulty with cooking creatively	Low (1–4.34)	40.8*	26.7	20.3	12.2
	Medium–low (4.35–5.67)	37.9*	20.2	21.3	20.6
	Medium–high (5.68–6.34)	19.7	20.2	30.7	29.4*
	High (>6.34)	16	18.6	30.5	34.9*
No difficulty with accurate planning	Low (1–4.34)	40.8*	26.7	20.3	12.2
	Medium–low (4.35–5.67)	37.9*	20.2	21.3	20.6
	Medium–high (5.68–6.34)	19.7	20.2	30.7	29.4*
	High (>6.34)	16	18.6	30.5	34.9*
Knowledge of prolonging shelf life	Low (1–4.0)	49.1*	20.1	19	11.8
	Medium–low (4.1–5.0)	22.7	24.8	28.4	24
	Medium–high (5.1–6.0)	18.2	22	27.4	32.4*
	High (>6.0)	19.9	16.2	29.8	34*
**Education received from parents**					
Parents’ attention to preventing food waste	Low (1–4.67)	51.1*	21.3	17.2	10.3
	Medium–low (4.7–5.67)	25.9	28.1	24.5	21.5
	Medium–high (5.68–6.9)	19.4	19.9	29.9	30.8*
	High (7.0)	16.9	18.7	30.7	33.7*

*p < 0.05 calculated performing the Chi-square test with Bonferroni correction.

As reported in Table 1, consumers with the highest AIDGI reported a medium–high and high capacity in assessing food safety (28.8 and 34.7%, *p* < 0.001), in planning accurately (29.4 and 34.9%, *p* < 0.01), in knowing how to prolong the shelf life of a product (32.4 and 34%, *p* < 0.05), and in cooking creatively (29 and 32%, *p* < 0.01). On the other hand, more than one-third of the respondents who obtained the lowest AIDGI level less frequently adopted these practices (*p* < 0.001), especially as far as concerning the knowledge of the shelf-life of a product (49.1%).

Finally, half of the respondents with low AIDGI scores did not receive any education regarding food waste (51.1%, *p* < 0.001) while those who achieved the highest AIDGI had a family in which the parents paid attention to avoid food waste (medium–high 30.8% and high 33.7%, *p* < 0.01).

## 4 Discussion

Food waste, eating habits, and nutritional education are intrinsically linked in terms of public health, environmental protection, and sustainability goals. The main purpose of this paper was to evaluate consumer food waste behaviors and food habits to demonstrate if and how these aspects are correlated. To the best of our knowledge, this is the first study that investigated the relationship between consumers’ food waste habits and the quality of their diet in a representative sample of the Italian population. The added value of this work is the interdisciplinary approach combining nutrition and sustainability aspects focusing on an emerging topic as food waste. In fact, according to Conrad et al. (22), it is necessary to collect more data concerning the relationship between the quality of the diet and food waste, since at the moment this is a critical research gap in the field of the sustainability of the food system.

The most significant result of this assessment is the strong statistically significant association between food waste attitudes and adherence to nutrition recommendations. The results of the contingency analysis confirmed that in the Italian consumers it is possible to demonstrate a polarization. In particular, among the group of population with higher AIDGI levels, higher HFWB indicators’ scores corresponding to behaviors aimed at preventing and limiting food waste were observed. On the other hand, in the lower AIDGI classes was reported a tendency to have lower HFWB scores that correspond to habits that may produce most waste. In other words, consumers with better food consumption patterns were also consumers with increased attention toward food waste in terms of prevention, knowledge of the problem, and abilities in the kitchen to limit the food that is thrown away. This is an important point in terms of policy and intervention strategies. In fact, according to our results, adherence to dietary recommendations is a driver of food waste prevention. These results represent an important answer to the prefixed research questions, hence demonstrating the importance of including food waste prevention practices in the framework of educational nutritional activities.

The correlation between food habits, through the AIDGI, and food waste behavior in Italy strengthens the concept that sustainability is a goal that can be achieved by combining different aspects. Although it was conducted in the UK and included participants aged only between 18 and 35 years old, the study developed by Savelli et al. (25) supports our findings and conclusions. The promotion of a healthy diet together with campaigns for food waste reduction could be public health actions that efficiently reach different population targets. It should be considered that in recent years, consumers have become more sensitive toward the issue of the sustainability of dietary choices (33, 34). Considering the HFWB indicators, the education received from parents on preventing food waste was particularly correlated to a high AIDGI. This finding could be interpreted considering that the acquisition of healthy eating habits, since childhood, could influence other aspects of sustainability including the attention to the limitation of food waste. It is a consolidated concept that educational strategies are most efficient if applied among the youngest. In various studies, educational activities targeting students resulted in positive behaviors changes, such as an increase in the consumption of fruit and vegetables and a decrease in the amount of food waste (35), or the adoption of a healthier lifestyle that includes sustainable food choices (locally grown or organic foods) (36, 37).

In addition to that, it is important that educational actions would reach the most in need. According to our data, the segment of the population with low AIDGI is also the group of people with a low level of indicators of food waste prevention and reduction behaviors. This population group should be identified as a target for educational campaigns combining nutrition and food waste topics.

The relationships between diet and food waste are complex. Conrad et al. and Carroll et al. (22, 23) found a correlation between higher levels of a certain type of food wasted (vegetables, fruit, dairy products) and a higher-quality diet. This could be expected considering quantitative aspects, since healthy eating means, among other aspects, a high level of consumption of fresh and perishable products impacting the quantity of discarded food. In addition to that Garnett et al. (38) described a scenario in which consumers reported knowledge, attention, and consideration in terms of sustainability even with difficulties in adopting behaviors in line with the intentions. This could be related either to the fact that consumers are resistant in changing their habits or to the ineffectiveness of current educational programs that probably should be more incisive.

Half of the sample reported a consumption pattern with low adherence to nutritional recommendations in particular among men, the youngest, and people living in large families. The homogenous distribution of the four levels of AIDGI found in Italy in this study was the same that was found in the previous study that used AIDGI in a selected sample of the population. As shown by Scalvedi et al. (30), categorizing AIDGI at three levels, the results were 32% low, 40% medium, and 28% high with a quite homogeneous third-party distribution. Even though improving the adherence to the Italian dietary guidelines, especially among the above-mentioned subgroups, is a necessary public health action, overall Italian eating habits showed interesting results in terms of the consumption of foods impacting the environment and influencing the sustainability of the dietary patterns. The quota of consumers that declared a low intake of red meat and adequate frequencies of consumption of white meat, fish, and plant-based products should be taken into account. The overtime changes in these consumption patterns need to be confirmed by an update of Italian food consumption data that presently are available for the period 2005–2006 (39). It is also true that these food habits combine health-promoting aspects with environmental issues. The recommendation of a consumption pattern that includes foods that preserve human health and natural resources was one of the main objectives of the last updated revision of the Italian dietary guidelines (19). These guidelines provide recommendations aligned with the most recent evidence not only on healthy eating but also on the wider social and environmental implications of dietary choices with the idea of promoting a food environment that contributes to good public and personal health, as well as to local and global environmental sustainability (40).

In addition, our results highlighted that most the Italian consumers tried to adopt behaviors aimed at preventing and reducing food waste during their daily life. Attention was paid to avoiding generating food surplus, or otherwise storing them, and then consuming the leftovers. This result was confirmed in the assessment of Scalvedi and Rossi (11) in which the attention of Italian consumers toward food waste, the habits of consuming all foods that are cooked and using the leftovers was already reported. Furthermore, similar data were reported by Annunziata et al. (41) in an assessment of food waste behaviors in Southern Italy in which the sub-sample that wasted less reported reusing leftovers more than the group that wasted higher quantities of food. Another aspect largely reported by Italian consumers is the influence of education from parents as a determinant of food waste preventive attitude. According to van Geffen et al. (7) awareness of parents for food waste prevention during the upbringing did not affect food waste levels directly. However, a higher awareness during the upbringing led to a better overview of the stored food, to cook precisely and to use leftovers, confirming the results of the present assessment.

The results of this study need to be interpreted also in consideration of the period of data collection. Even though it was realized after the lockdown period–that in Italy was gradually reduced from the 18th of May 2020–the pandemic could have influenced dietary habits and food waste behaviors. The effect of the COVID-19 pandemic had an impact on dietary practices both negatively and positively throughout Europe. Several studies reported an increase in the quality of the diet with increased consumption of healthy foods that were, however, associated with poor lifestyle outcomes such as weight gain and limited physical activity (42, 43). In terms of food waste, in a survey carried out during the most restrictive phase of the containment measures against COVID-19 in Italy, the awareness of food surplus and waste was reported by nearly 80% of participants who claimed to have consumed all the food they cooked and reported to have had the capacity to store surplus and consume the leftovers (44). The consumers’ awareness toward food waste during the pandemic period was confirmed by other European studies (45–47). According to Principato et al. (48), in Italy, the unexpected positive effect of the lockdown caused by the pandemic was that most consumers threw away less food in comparison to the pre-pandemic situation with a better implementation of food management practices (shopping list, meal planning, etc.). In addition, the logistical difficulties of grocery shopping experienced by consumers resulted in an increased capacity for handling household food consumption, with a reduction in the amount of food wasted. In light of these points, our results on the avoidance of impulse buying together with the reported cooking creativity could be the carryover effect of the lockdown period, after which this data collection was realized. Further assessments are needed to monitor the trends to detect changes in food waste behavior.

This study has strengths and limitations. The strength of this work is represented by the sampling methodology that provided the national representativeness of the Italian adult population in terms of gender, age, income, and education. Another important added value of this assessment is the use of a questionnaire that had already been tested in Italy. The questionnaires were specifically designed to collect information on HFWB and AIDGI as the main outcomes of the study, in line with the pre-determined objectives. However, this kind of study has a general limitation related to self-reported answers that could affect the reliability of the responses. The food waste behavior and the eating habits assessed were based on the participants’ perceptions that may not reflect reality and the answers could be influenced by the willingness of declaring behaviors corresponding to socially desirable norms or healthy food consumption practices. However, the large sample size, the robustness of the methodology, and the confirmation of our results with other similar surveys support the reliability of the data collected.

In conclusion, the achievement of SDGs includes the improvement of human health guaranteeing access to healthy foods for all, and the reduction of food waste at the consumer level. With this study, we demonstrated that in Italy these aspects are correlated. Food waste is associated with nutrient wastage and food waste reduction interventions can successfully address food sustainability and nutrition quality. According to the present paper, Italians that follow nutritional recommendations are also consumers with higher attention toward the limitation of food waste. This is a point to take into consideration while planning nutrition education actions.

Food waste prevention and reduction are key aspects of sustainability and a responsible food consumption attitude. According to Springmann et al. (49), the inclusion of sustainability of food choices into the nutritional recommendations could be not only beneficial from a health perspective but also necessary to meet global sustainability goals and to stay within the environmental limits of the food system. The results of the present study are in line with these points.

Considering the scarcity of data collected concerning AIDGI and HFWB at the national level, this study could be considered a benchmark for future monitoring assessments despite the exceptional events that took place in 2020 due to the COVID-19 pandemic. This data confirmed the importance of targeting the younger age groups who are most in need of nutrition education actions. The recent increase in young people’s awareness of climate and environmental issues could be exploited to transmit the message regarding the importance of combining healthy food behavior and food waste issues as key elements to improve the sustainability of their dietary choices.

## Data availability statement

The raw data supporting the conclusions of this article will be made available by the authors, without undue reservation.

## Ethics statement

Ethical review and approval was not required for the study on human participants in accordance with the local legislation and institutional requirements. The patients/participants provided their written informed consent to participate in this study.

## Author contributions

FG and LR contributed to the conceiving, writing, and reviewing the manuscript. LR was responsible for the overall supervision, project administration, and funding acquisition. Both authors read and agreed with the published version of the manuscript.
