# Supplementary material for: Sustainable choices: The relationship between adherence to the dietary guidelines and food waste behaviors in Italian families
Source: Front Nutr. 2022 Dec 14;9:1026829. doi: 10.3389/fnut.2022.1026829 (PMC9794859; doi:10.3389/fnut.2022.1026829)
Supplement: Supplementary file 1 [file Table_1.DOCX]

Table S1. The complete questionnaire.

| **Questions** | **Answers** |
| --- | --- |
| **PERSONAL DATA** | |
| **Gender** | Male/Female |
| **Age (years old)** | 18-24 /25-34 /35-44 /45-54 /55-64 /> 64 |
| **Geographical area of residence (regions and macro-regions)** | Alpina area regions (Piemonte, Liguria, Valle D'Aosta)/Lombardia region  Northwest area regions (Trentino Alto Adige, Friuli Venezia Giulia)/Veneto region  Emilia-Romagna region/ Apennine Area regions (Toscana, Umbria)/Adriatic area regions (Marche, Abruzzo)/Lazio |
| **Education level** | Without qualification/primary school/middle school/high school/professional institute diploma/high school diploma/university in progress/university diploma/bachelor degree/master degree/after degree specialization/PhD |
| **Job** | Self-employed/employee(office worker or highly specialized worker)/student/seeking the first job/unemployed/housewife/retired from work / pensioner |
| **Income** | up to 18.000 euros/between 18.001 and 27.000 euros/between 27.001 and 36.000 euros/between 36.001 and 54.000 euros/between 54.001 and 72.000 euros/between 72.001 and more/I prefer to not answer |
| **Family size** | 1 person/2 people/3 people/4 people/5 or more people |
| **SECTION 1- Household food waste behaviors** | |
| ***Household food waste prevention practices***  **Planning the shopping and using the food**  I plan what to cook for each day of the week/I usually eat foods that are going to be spoiled/I plan the handling of food in the household/I plan what to buy and what to cook/I do the shopping list  **Avoiding impulsive buying**  Regarding food, I consider myself an impulsive buyer/I buy food that I have not plan to buy/I buy products that I do not need  **The overview of stored food**  My shelves and/or fridge are organized/I make sure that food that needs to be eaten first lies in sight/I can see what I stored in one glance/I know exactly what I stored  **Cooking the right quantities of food**  I try to not have any unnecessary leftovers/I usually measure the ingredients of the meal/I am precise to cook the right quantities/ Before cooking, I think carefully about the quantities I need  **Storing and using leftovers**  I finish food that was served/If I serve too much food, I save the leftovers/If I cook too much food, I save the leftovers/The leftovers I stored will be eaten/All food that I prepared will eventually be eaten (including leftovers)/If I have leftovers on the plate or in the pan, they will be stored | **Never; rarely; occasionally; sometimes; frequently; usually; always** |
| ***Abilities***  **The perceived difficulty with assessing food safety**  I find difficulties with estimating if food is still safe to eat using smell or taste/It is difficult to estimate if food is still safe to eat/ Sometimes I do not know if food is still safe to eat  **The perceived difficulty with cooking creatively**  I find difficulties with preparing a meal with products I have at home/I find difficulties with cooking with leftovers/I find difficulties with using a recipe that I do not know/I leave foods in the fridge, because I do not know how to cook them  **The perceived difficulty with an accurate planning**  I find difficulties with estimating how much food I should buy /I find difficulties with estimating how much food my family should eat during the week/I find difficulties with estimating how much food I should cook for the main meal  **The knowledge of prolonging the shelf-life**  I know the best way to keep fruit and vegetables fresh as long as possible/I know the best way to keep meat and fish fresh as long as possible/I know the fridge temperature to keep my food fresh as long as possible/I know how to keep products fresh as long as possible | **Strongly disagree; quite disagree; neither disagree nor agree; quite agree; agree; strongly agree** |
| ***Education received from parents***  **Parents’ attention to prevent food waste**  My parents paid attention to prevent food waste/My parents taught me to handle food with appreciation and care/When growing up, I was not allowed to throw away food | **Strongly disagree; disagree; quite disagree; neither disagree nor agree; quite agree; agree; strongly agree** |
| **SECTION 2- Eating habits-Frequency of food consumption** | |
| **Eggs/red meat/potatoes/savory snacks/fish and fisheries products/sugary drinks/ white meat/processed and cured meat/legumes/cakes and sweet snacks/nuts/dairy products/beer and wine/ other alcoholic drinks/milk and yogurt/vegetables/bread, pasta, rice/fresh fruits** | **More than once per day; once per day; few times per week; less than once a week; never** |
