# Supplementary material for: Sustainable choices: The relationship between adherence to the dietary guidelines and food waste behaviors in Italian families
Source: Front Nutr. 2022 Dec 14;9:1026829. doi: 10.3389/fnut.2022.1026829 (PMC9794859; doi:10.3389/fnut.2022.1026829)
Supplement: Supplementary file 2 [file Table_2.DOCX]

| **Gender** | **(%)** |  |  | **Family size** | **(%)** |
| --- | --- | --- | --- | --- | --- |
| **Women** | 52 |  |  | 1 person | 13 |
| **Men** | 48 |  |  | 2 people | 34 |
|  |  |  |  | 3 people | 25 |
| **Age** |  |  |  | 4 people | 22 |
| **18-24** | 8 |  |  | 5 or more people | 7 |
| **25-34** | 13 |  |  |  |  |
| **35-44** | 16 |  |  | **Household income (monthly)** |  |
| **45-54** | 19 |  |  | up to 18,000 euros | 21 |
| **55-64** | 17 |  |  | between 18,001 and 27,000 euros | 24 |
| **> 64** | 27 |  |  | between 27,001 and 36,000 euros | 19 |
|  |  |  |  | between 36,001 and 54,000 euros | 15 |
| **Education level** |  |  |  | between 54,001 and 72,000 euros | 5 |
| **Low** | 49 |  |  | between 72,001 and more | 4 |
| **Medium** | 36 |  |  | I prefer not to answer | 13 |
| **High** | 15 |  |  |  |  |
|  |  |  |  | **Geographical area of residence (regions and macro-regions)** |  |
| **Job** |  |  |  | Alpina area regions (Piemonte, Liguria, Valle D'Aosta) | 10 |
| **Self-employed** | 14 |  |  | Lombardia region | 17 |
| **Employee** | 35 | Office worker | 75 | Northwest area regions (Trentino Alto Adige, Friuli Venezia Giulia) | 4 |
|  |  | Highly specialized workers | 25 | Veneto region | 8 |
| **Student** | 6 |  |  | Emilia-Romagna region | 7 |
| **Seeking first job/ unemployed** | 9 |  |  | Apennine Area regions (Toscana, Umbria) | 8 |
| **Housewife** | 12 |  |  | Adriatic area regions (Marche, Abruzzo) | 5 |
| **Retired from work / pensioner** | 23 |  |  | Lazio | 10 |
|  |  |  |  | Southeast area regions (Puglia, Molise) | 7 |
|  |  |  |  | Campania region | 9 |
|  |  |  |  | Southwest area regions (Calabria, Basilicata) | 4 |
|  |  |  |  | Islands (Sicilia, Sardegna) | 11 |

Table S2. The sociodemographic characteristics of the sample.
