# Supplementary material for: Sustainable choices: The relationship between adherence to the dietary guidelines and food waste behaviors in Italian families
Source: Front Nutr. 2022 Dec 14;9:1026829. doi: 10.3389/fnut.2022.1026829 (PMC9794859; doi:10.3389/fnut.2022.1026829)
Supplement: Supplementary file 3 [file Table_3.DOCX]

|  | More than once per day  (%) | Once per day  (%) | Few times per week  (%) | Less than once a week  (%) | Never  (%) |
| --- | --- | --- | --- | --- | --- |
| Fresh fruit | 40.7 | 30.4 | 20.7 | 6.1 | 2 |
| Vegetables | 29.8 | 35.6 | 28.4 | 4.6 | 1.6 |
| Bread, pasta, rice | 19.9 | 49.8 | 24.8 | 3.8 | 1.6 |
| Milk and yogurt | 10 | 41.1 | 27.7 | 12.2 | 9 |
| Dairy products | 3.9 | 20.1 | 57.8 | 13.9 | 4.3 |
| Eggs | 1.8 | 7.6 | 58.3 | 29.7 | 2.7 |
| Nuts | 6.1 | 17.2 | 34.7 | 33.5 | 8.5 |
| Legumes | 4.1 | 15 | 55.9 | 21.1 | 3.9 |
| Processed and cured meat | 2.7 | 13.8 | 53.4 | 25.2 | 4.9 |
| Poultry | 2.5 | 13.6 | 68.3 | 11 | 4.5 |
| Fish and fisheries products | 2.2 | 9.9 | 55.4 | 27.1 | 5.4 |
| Potatoes | 2 | 8.1 | 53 | 33.9 | 3 |
| Savory snacks | 2.5 | 8.3 | 32 | 41.5 | 15.7 |
| Cakes and sweet snacks | 4.2 | 15.3 | 40.7 | 34.4 | 5.4 |
| Red meat | 2 | 7.7 | 52.4 | 31.9 | 6 |
| Beer and wine | 7.2 | 19.4 | 29.1 | 24.7 | 19.6 |
| Other alcoholic drinks | 1.9 | 7.8 | 20.3 | 33.9 | 36.1 |
| Sugary drinks | 3.1 | 11.1 | 26.9 | 33.7 | 25.3 |

Table S3. Food habits of the Italian families. The color of the cells corresponds to the Adherence to the Italian Dietary Guidelines Indicator (AIDGI) score: white, score = 0; light gray, score = 1; dark gray, score = 2*.*
