# Supplementary material for: Sustainable choices: The relationship between adherence to the dietary guidelines and food waste behaviors in Italian families
Source: Front Nutr. 2022 Dec 14;9:1026829. doi: 10.3389/fnut.2022.1026829 (PMC9794859; doi:10.3389/fnut.2022.1026829)
Supplement: Supplementary file 4 [file Table_4.DOCX]

Table S4. The relationship between AIDGI and sociodemographic variables; * p<0.05 calculated performing the Chi-square test with Bonferroni correction.

|  | | **AIDGI levels** | | | |
| --- | --- | --- | --- | --- | --- |
| **OVERALL SAMPLE** | | Low  (%)  28.9 | Medium-low  (%)  21.5 | Medium-high  (%)  25.5 | High  (%)  24.1 |
| **GENDER** | Male | 34.3* | 24.1* | 23.4 | 18.2 |
|  | Female | 23.8 | 19 | 27.5* | 29.6* |
| **AGE GROUP** | 18-24 years | 43.6* | 22.1 | 20.1 | 14.2 |
|  | 25-34 years | 38.9* | 25.8 | 20.5 | 14.7 |
|  | 35-44 years | 42.7* | 21.4 | 20.7 | 15.2 |
|  | 45-54 years | 26.4 | 24.6 | 26.8 | 22.3* |
|  | 55-64 years | 24.7 | 19.2 | 26.7 | 29.4* |
|  | >64 years | 15.8 | 18.4 | 30.8 | 34.9* |
| **FAMILY SIZE** | 1 person | 27.2 | 25 | 23.5 | 24.2 |
|  | 2 people | 24.8 | 17.6 | 28.3 | 29.3* |
|  | 3 people | 31.6 | 22.6 | 23.5 | 22.3 |
|  | 4 people | 28.8 | 24.5 | 26.3 | 20.4 |
|  | ≥5 people | 42.3* | 21.2 | 20.7 | 15.8 |
